# The coevolution of sexual imprinting by males and females

**DOI:** 10.1002/ece3.2409

**Published:** 2016-09-14

**Authors:** Miguel Angel Gómez‐Llano, Eva María Navarro‐López, Robert Tucker Gilman

**Affiliations:** ^1^ School of Earth and Environmental Science The University of Manchester Manchester UK; ^2^ School of Computer Sciences The University of Manchester Manchester UK

**Keywords:** evolutionary stable strategies, learning, mathematical model, sexual selection

## Abstract

Sexual imprinting is the learning of a mate preference by direct observation of the phenotype of another member of the population. Sexual imprinting can be paternal, maternal, or oblique if individuals learn to prefer the phenotypes of their fathers, mothers, or other members of the population, respectively. Which phenotypes are learned can affect trait evolution and speciation rates. “Good genes” models of polygynous systems predict that females should evolve to imprint on their fathers, because paternal imprinting helps females to choose mates that will produce offspring that are both viable and sexy. Sexual imprinting by males has been observed in nature, but a theory for the evolution of sexual imprinting by males does not exist. We developed a good genes model to study the conditions under which sexual imprinting by males or by both sexes can evolve and to ask which sexual imprinting strategies maximize the fitness of the choosy sex. We found that when only males imprint, maternal imprinting is the most advantageous strategy. When both sexes imprint, it is most advantageous for both sexes to use paternal imprinting. Previous theory suggests that, in a given population, either males or females but not both will evolve choosiness in mating. We show how environmental change can lead to the evolution of sexual imprinting behavior by both sexes in the same population.

## 1 Introduction

Sexual imprinting is a form of learned mate preference for a trait that an individual has observed in its population. Imprinted preferences are acquired during the early stages of life and influence adult mate choice and pair formation (Immelmann, 1975). Sexual imprinting is common and widely distributed in animals. It is found in more than 100 species of birds (ten Cate & Vos, 1999; Verzijden et al., 2012), as well as in insects (Westerman, Hodgins‐Davis, Dinwiddie, & Monteiro, 2012), spiders (Hebets, 2003), fishes (Kozak & Boughman, 2009; Kozak, Head, & Boughman, 2011; Verzijden et al., 2008), and mammals (Kendrick et al., 1998), possibly including humans (Marcinkowska & Rantala, 2012; Rantala & Marcinkowska, 2011).

A large research effort, both empirical and theoretical, has explored the evolutionary consequences of sexual imprinting. Sexual imprinting can push genotypes to fixation (Aoki et al., 2001; Laland, 1994a) or cause runaway selection of traits that carry viability costs (Ihara & Feldman, 2003; Laland, 1994b). Sexual imprinting can also create barriers to gene flow, allowing reproductive isolation to emerge between populations (Bateson, 1978; Dukas, 2006; Gilman & Kozak, 2015; Grant & Grant, 1996; Laland, 1994a; ten Cate & Bateson, 1988, 1989; Verzijden et al., 2005; Verzijden et al., 2012).

Different mechanisms have been hypothesized to explain the evolution of sexual imprinting. For example, by helping individuals to acquire preferences for mates of their own species, sexual imprinting might allow individuals to avoid the costs of heterospecific mating (Immelmann, 1972; Irwin & Price, 1999). In sexually dimorphic species, imprinting might facilitate sex identification (ten Cate & Vos, 1999) and prevent individuals from wasting time and energy courting same‐sex partners (Banerjee & Adkins‐Regan, 2014; Immelmann, 1975). Sexual imprinting might also help individuals to choose partners that offer high‐quality parental care to their offspring (Little et al., 2011). In this study, we will focus on one particular mechanism that is likely to contribute to the evolution of mate preferences: the good genes mechanism (Moller & Alatalo, 1999). The good genes mechanism hypothesizes that sexual imprinting allows individuals to choose mates that carry fit alleles, and therefore will pass fit alleles to the offspring of the choosy individual. Much of the previous work on the evolution of sexual imprinting has focused on the good genes mechanism (Chaffee et al., 2013; Invernizzi & Gilman, 2015; Tramm & Servedio, 2008), and a thorough understanding of this mechanism can serve as a baseline for investigating other mechanisms that might promote the evolution of sexual imprinting.

Studies of how sexual imprinting evolves have focused on two properties of imprinting: the imprinting mode and the imprinting strength. Together, these comprise an imprinting strategy (Chaffee et al., 2013). The imprinting mode determines the set of individuals from which the preference was acquired (i.e., the imprinting set, Tramm & Servedio, 2008). Sexual imprinting is often classified into one of three modes. Individuals can imprint on traits of their fathers (paternal imprinting), their mothers (maternal imprinting), or other members of the parental generation (oblique imprinting) (Chaffee et al., 2013; Tramm & Servedio, 2008; Verzijden et al., 2005). The imprinting strength reflects the probability that a choosy individual rejects a potential mate with a phenotype different from that which the chooser has learned to prefer. Thus, imprinting strength measures choosiness.

Tramm and Servedio (2008) used pairwise comparisons of different imprinting modes with fixed imprinting strengths to understand which mode provides the greatest fitness advantage to choosy females. Their study explained the role of the imprinting set in the success of an imprinting strategy. Chaffee et al. (2013) used an adaptive dynamics framework where choosiness could evolve to analyze the conditions under which different imprinting modes are evolutionarily stable. They found that even small costs of building or maintaining imprinting apparatus (i.e., fixed costs, sensu Otto et al., 2008) can prevent the evolution of sexual imprinting. If the sensory and neurological apparatus necessary for imprinting is maintained for some other purpose (e.g., foraging), then imprinting can evolve. In such cases, paternal imprinting is the stable imprinting mode for females. This suggests that imprinting by females in nature should be paternal (Chaffee et al., 2013).

Past theoretical studies have assumed that only females are choosy (Chaffee et al., 2013; Invernizzi & Gilman, 2015; Tramm & Servedio, 2008), but in nature, males can also be choosy. Recent studies of male mate choice have led some authors to argue that male choosiness is far more common than previously believed (Amundsen, 2000; Bonduriansky, 2001). Behaviors such as cryptic male choice, defined as the variation in the amount of reproductive effort (e.g., sperm allocation and copulation time) allocated to different females (Bonduriansky, 2001), can make male mate choice difficult to detect and increase the misconception that males are not choosy (Engqvist & Sauer, 2001).

The evolution of mate choosiness by males has been studied (Barry & Kokko, 2010; Kokko & Johnstone, 2002; Servedio, 2007; Servedio & Lande, 2006). Using a mathematical model, Barry and Kokko (2010) predicted that males will be choosy when females are common, variability in the quality of females is high, and courtship is costly. This prediction matches empirical results. For example, Kvarnemo and Simmons (1999) found that male bush crickets are choosier when the operational sex ratio (sensu Emlen & Oring, 1977) is female‐biased and females vary in quality. Kvarnemo and Simmons argued that the high cost of missed mating opportunities prevents the evolution of male choosiness when sex ratios are male‐biased.

Sexual imprinting by males has been observed in nature (Bateson, 1978; Bereczkei et al., 2004; ten Cate & Vos, 1999; Kendrick et al., 1998; Vos, 1995a,b), and the imprinting strategies of males and females can differ in the same population. For example, zebra finch males (*Taeniopygia guttata*) imprint on the beak color of their mothers (Vos et al., 1993; Weisman et al., 1994) while females imprint on the beak color of their fathers (ten Cate et al., 2006; Vos, 1995b). To date, however, there have been no studies investigating the evolution of sexual imprinting in males or in both sexes in the same population.

Here, we study how sexual imprinting by males and by both sexes evolves, and we identify which sexual imprinting strategies are most likely to evolve under the good genes mechanism. We pose two main questions: (1) under a particular combination of imprinting mode, sex ratio, and cost of courtship, which sex should we expect to evolve imprinting and how strong should we expect imprinting to become? and (2) when all modes of imprinting are possible, which mode is most likely to evolve? To answer these questions, we first identify the evolutionarily stable strength of imprinting (i.e., the choosiness that cannot be invaded by different female or male choosiness strengths) for each imprinting mode across a range of sex ratios and costs of male courtship. Then, we identify the imprinting modes that, at their evolutionarily stable strengths, are stable against invasion by other modes. To our knowledge, this is the first study to analyze the evolution of male sexual imprinting and the interaction between male and female sexual imprinting.

## 2 The Population Genetic Model

### 2.1 Overview

**Figure 1:**
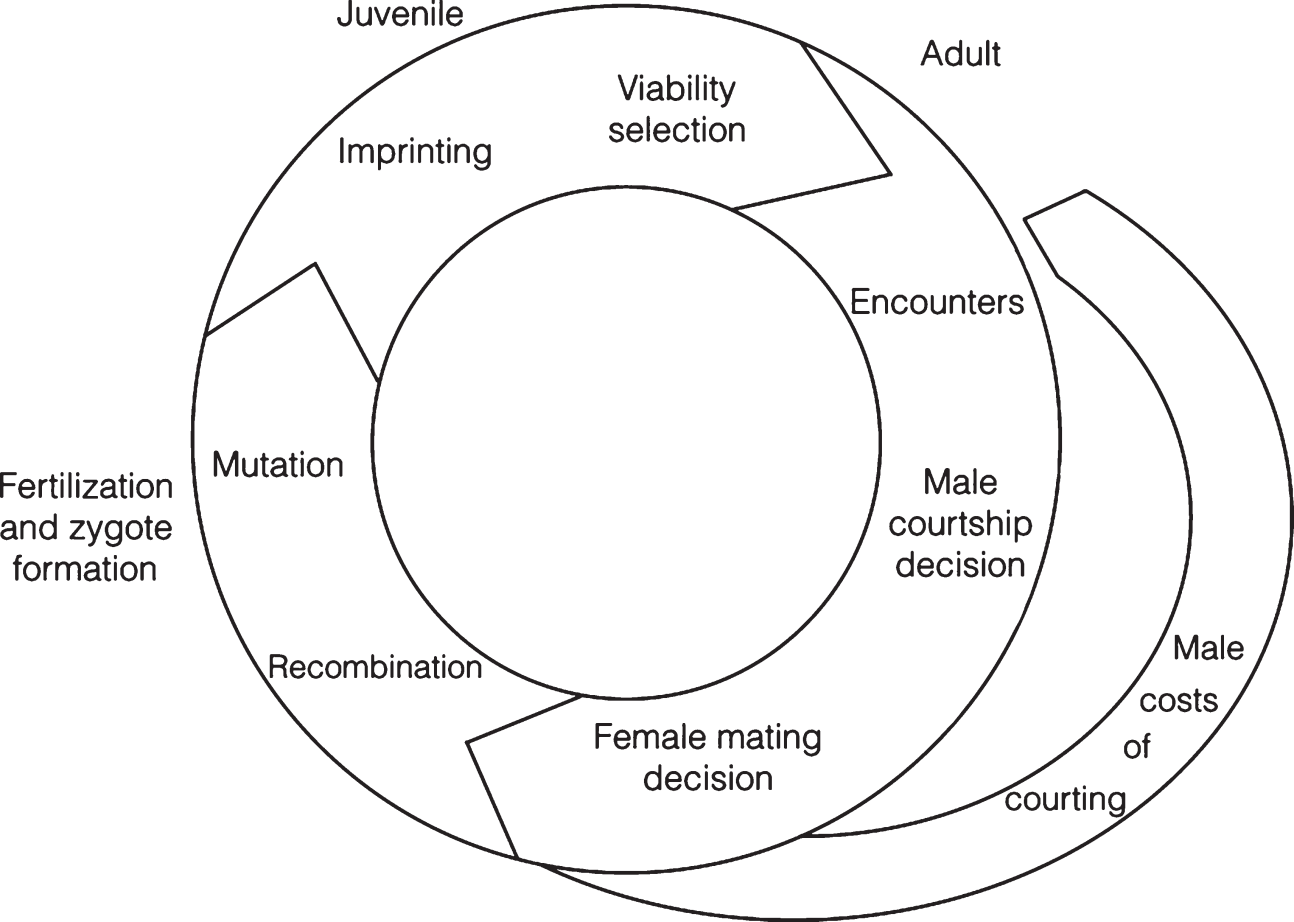
Schematic of the population dynamic model. Generations start with juveniles. Juveniles undergo viability selection. Surviving individuals become adults and enter the mating phase. In the mating phase, males encounter females. Males decide whether to court, and males that court incur a cost of courtship. Females decide whether to accept courting males. Males re‐enter the mating pool, but mated females leave the mating pool. After 20 rounds of mating, offspring are produced with free recombination and a low rate of mutation at the target trait locus. Offspring become juveniles in the next generation

We modeled a population with sexually monomorphic viability selection acting on a single genetically controlled “target” trait. Each generation begins with juveniles. Juveniles that survive viability selection become adults and enter the mating phase. The sex ratio of adults entering the mating phase is a parameter of the model. Mating occurs in rounds and is by mutual choice. In each round, females meet males at random. Males decide whether to court and females decide whether to accept the courting male, each according to an imprinted preference for a particular target phenotype. We assume a polygynous system where males can mate multiple times but females mate only once. Rejecting mates is potentially costly for males (which forgo mating opportunities) and for females (which can die without mating). Empirical studies show that courtship is energetically costly (Cordts & Partridge, 1996; Judge & Brooks, 2001; Kotiaho, 2001). We implement this cost in our model by assuming that males that court in one round are less able to enter into the mating pool in future rounds. As a result, the operational sex ratio in each mating round changes as females leave the mating pool due to mating and males leave the mating pool due to the cost of previous courtships. After mating, offspring form the next generation. Figure 1 summarizes the order of events in our model.

Sexual imprinting strategies in our model are genetically controlled and subject to mutation. Therefore, they can evolve. We modify the adaptive dynamics model of Chaffee et al. (2013) to find the sexual imprinting strategies that are evolutionary stable at different adult sex ratios and costs of courtship. Adaptive dynamics is a powerful tool for studying evolutionary trajectories under potentially weak selection (Dercole & Rinaldi, 2002), but it ignores stochasticity in evolution (Waxman & Gavrilets, 2005). Thus, our model predicts expected evolutionary trajectories for sexual imprinting, but does not predict the range or variability of possible evolutionary outcomes.

### 2.2 Population

We constructed a genetically explicit model of a haploid population of males and females. At any time, we are interested in only two diallelic loci in the genome. The first locus controls a trait that is the target of viability selection and is used by both sexes to assess potential mates. This locus houses allele T or t. We assume that the target trait is expressed equally in males and females, and therefore that individuals identify the correct sex for mating according to some other trait or set of traits. The second locus controls the imprinting strategy and houses alleles S or s. An imprinting strategy comprises both an imprinting mode (maternal, paternal, or oblique) and a strength of choosiness. The same genotype may confer different imprinting strategies in males and females (e.g., males might imprint on mothers and females on fathers). We assume that S and s differ in only one aspect of the imprinting strategy (i.e., they differ in male imprinting mode, male choosiness, female imprinting mode, or female choosiness). Biologically this is reasonable if the imprinting strategy is controlled by many loci, but mutation is rare enough that only one locus is polymorphic at any time. This extends a standard assumption of adaptive dynamics (Geritz et al., 1997). We assume that neither the S nor s allele has a direct effect on viability (i.e., there is no fixed cost of choosiness). The effect of fixed costs on the evolution of sexual imprinting has been studied elsewhere (Chaffee et al., 2013).

Each individual in the population has a nongenetic imprinted phenotype, P or p. This phenotype is a preference for mates with one of the target trait alleles. Individuals with phenotype P prefer mates with target trait allele T and those with phenotype p prefer mates with target trait allele t. Individuals acquire the P or p phenotype according to their imprinting strategies. For example, a paternally imprinting individual whose father has the T genotype acquires the P phenotype.

Each individual in the population is fully characterized by its sex and its phenogenotype (i.e., STP, STp, StP, Stp, sTP, sTp, stP, and stp). We assume that the population is large enough that we can ignore demographic stochasticity. Therefore, we can monitor the population by tracking only the frequencies of the eight male and eight female phenogenotypes.

### 2.3 Dynamics

For simplicity, we assume that the population undergoes discrete generations. The population experiences two distinct selection pressures: viability selection during the juvenile stage and sexual selection during the mating phase. Viability selection determines the proportion of each phenogenotype that survives to the mating phase, and sexual selection determines the proportion that reproduces. The offspring of the successful phenogenotypes form the next generation.

#### 2.3.1 Viability selection

We define the t allele as the less fit allele. In particular, the t allele confers a phenotype that experiences a negative viability effect of *v*_*t*_. Let *M*_*ijk*_ represent the frequency of males with imprinting strategy*i* ∈ {S,s}, target trait *j* ∈ {T,t}, and imprinted preference *k* ∈ {P,p}. Let *v*_*j*_ represent the viability cost of trait *j*. The frequency of males with the phenogenotype *ijk* after viability selection is

(1) $$M_{ijk}^{\prime }=\frac{\left(1-v_{j}\right)M_{ijk}}{\sum_{xyz}\left(1-v_{y}\right)M_{xyz}}$$

The numerator in eq. (1) captures the effect of viability selection, and the denominator scales the male component of the population back to unity. We calculate $F_{ijk}^{\prime }$, the density of females with phenogenotype *ijk* after viability selection, with the analogous equation. In the body of the study, we assume that *v*_*t*_ = 0.01 and *v*_*T*_ = 0 in both sexes. Thus, viability selection is weak and monomorphic. In Appendix 1, we present results when the t allele has different viability effects in the two sexes, and in Appendix 2, we present results for strong selection (i.e., *v*_*t*_ = 0.9). In each case, results are qualitatively similar to those we present here.

We controlled the sex ratio in the adult population with a parameter α. Specifically, the density of males of each phenogenotype reaching adulthood is:

(2) $$M_{ijk}^{\prime \prime }=\alpha M_{ijk}^{\prime }$$

Thus, at adulthood, there are α males for each female in the population. Biased sex ratios might be due to the sex ratio at birth or to different survival rates in the sexes, but in our model, they are not due to viability selection on the target trait.

#### 2.3.2 Mating

In the mating phase, mating opportunities are sequential and occur in rounds. In each round, males encounter females at random, independent of phenogenotype. Encounters are limited by the availability of the rarer sex in the mating pool. The encounter probability for the rarer sex is always one, even if the density of both sexes becomes low. This ensures that individuals have many mating opportunities, reduces the cost of rejecting potential mates, and promotes the evolution of choosiness (Kokko & Johnstone, 2002; Kokko & Monaghan, 2001). Results when the encounter rates for both sexes decrease with the density of individuals remaining in the mating pool are qualitatively similar (Appendix 3). Mating continues for 20 rounds, after which unmated individuals die without mating. This creates a relative cost (sensu Otto et al., 2008) of choosiness. The density of encounters between males with phenogenotype *ijk* and females with phenogenotype *xyz* in mating round *r* is

(3) $$E_{xyz}^{ijk}\left(r\right)=\frac{\left(M_{ijk}^{\prime \prime }\left(r\right)\right)\left(F_{xyz}^{\prime }\left(r\right)\right)}{N(r)},$$

where *N*(*r*) is the density of the more common sex in the mating pool.

When a male encounters a female, he decides whether to court according to his preferred mate phenotype and his choosiness. A male always courts a female with his preferred phenotype. He courts a female with the opposite phenotype with probability 1/*b*_*i*_, where *b*_*i*_ is the choosiness of a male with imprinting strategy *i*. If the male courts, then he incurs a cost of courtship. We implement this cost by assuming that each courtship reduces the ability of the male to seek mates in future mating rounds by some proportion *c*. This might be true if courtship is costly in terms of time or energy. If the male courts, then the female decides whether to accept him according to her preferred phenotype and her choosiness. A female always accepts a courting male with her preferred phenotype and accepts a male with the opposite phenotype with probability 1/*a*_*x*_, where *a*_*x*_ is the choosiness of a female with imprinting strategy *x*. Thus, for both males and females, the greater the choosiness, the less likely an individual is to accept a mate with a nonpreferred phenotype.

Let $R_{xyz}^{ijk}\left(r\right)$ be the density of matings between males with phenogenotype *ijk* and females with phenogenotype *xyz* in round *r* of mating. Let *B*_*iyk*_ be the probability that a male with imprinting strategy *i* and imprinted preference *k* courts a female with target trait phenotype *y*, and let *A*_*xjz*_ be the probability that a female with imprinting strategy *x* and imprinted preference *z* accepts a courting male with target trait phenotype *j*. Thus, *B*_*iyk*_ *= *1 if *y = *T and *k =* P or if *y = *t and *k = *p (i.e., the female trait matches the male preference) and *B*_*iyk*_ *= 1/b*_*i*_ otherwise, and *A*_*xjz*_ *= *1 if *j = *T and *z =* P or if *j = *t and *z = *p (i.e., the male trait matches the female preference) and *A*_*xjz*_ *= 1/a*_*x*_ otherwise. Then,

(4) $$R_{xyz}^{ijk}\left(r\right)=E_{xyz}^{ijk}\left(r\right)B_{iyk}A_{xjz}$$

Females mate only once, and after this, they are removed from the pool of potential mates. Thus, the density of females with phenogenotype *xyz* in mating round *r *+* *1 is

(5) $$F_{xyz}^{\prime }\left(r+1\right)=F_{xyz}^{\prime }\left(r\right)-\sum_{ijk}R_{xyz}^{ijk}\left(r\right)$$

The first element in eq. (5) captures the density of females in round *r*, and the second element subtracts the females that mate in that round. Males can mate more than once, and the density of males in each mating round declines as the cumulative cost of previous courtships increases. The density of males with phenogenotype *ijk* in the mating pool in round *r *+* *1 is

(6) $$M_{ijk}^{\prime \prime }\left(r+1\right)=M_{ijk}^{ii}\left(r\right)-\sum_{xyz}E_{xyz}^{ijk}\left(r\right)B_{iyk}c$$

In eq. (6), the first element captures the density of males in round *r*, and second element adjusts for the cost of courtship in that round.

The relative density of matings between males with genotype *ij* and females with genotype *xy* over all rounds of mating is:

(7) $$R_{xy}^{ij}=\frac{\sum_{zk}\sum_{r=1}^{20}R_{xyz}^{ijk}\left(r\right)}{\sum_{\eta \iota \kappa }\sum_{\tau \upsilon \omega }\sum_{r=1}^{20}R_{\tau \upsilon \omega }^{\eta \iota \kappa }\left(r\right)}$$

The numerator in eq. (7) collects all of the ways a mating between males of genotype and *ij* females of genotype *xy* can arise, and the denominator scales the total density of matings in the population to unity.

All females that mate produce the same expected number of offspring, regardless of phenogenotype. Each offspring inherits a target trait and an imprinting allele from its parents, with free recombination. Thus, an individual can inherit both alleles from one parent or one allele from each parent. Each target trait allele mutates to the opposite allele with probability μ = 10^−6^. Finally, each offspring acquires an imprinted phenotype according to its mode of imprinting. In the case of oblique imprinting, the probability that an offspring acquires the P phenotype is equal to the frequency of the T allele among all adults in the parental generation. Offspring form the population in the next generation.

## 3 Analysis of Evolutionary Dynamics

Our goal was to understand how evolutionary stable strategies (ESSs) (Geritz et al., 1997; Maynard Smith & Price, 1973) for sexual imprinting depend on courtship costs and sex ratios. We began by finding the locally stable imprinting strength (i.e., choosiness) for each combination of imprinting modes (i.e., one mode used by males and one mode used by females) under different combinations of courtship cost and sex ratio. We allowed males and females to have different choosinesses (i.e., male and female choosiness evolves separately). Then, we tested whether the locally stable choosiness is stable against all other choosinesses in the same mode combination (i.e., whether the locally stable choosiness is also globally stable within its mode combination). Finally, we asked whether imprinting strategies with globally stable choosinesses are stable when invaded by imprinting strategies with different modes.

### 3.1 Finding locally stable imprinting strengths

To find the locally stable imprinting strengths, we modified the adaptive dynamics approach of Chaffee et al. (2013). We constructed adaptive landscapes (Gavrilets, 2004; Wright, 1932) with dimensions corresponding to male and female choosiness for different combinations of male and female imprinting mode, courtship cost, and sex ratio. We studied male and female imprinting modes in the set {maternal, paternal, oblique}, courtship costs in the set *c *= {0, 0.1, …,0.9}, and sex ratios in the set α = 10^i/9^ for i∈{−9,−8,…,9} (i.e., adult male‐to‐female sex ratios range from 1:10 to 10:1). The fitness peaks of these landscapes represent imprinting strategies that cannot be invaded by small mutations to choosiness.

Because the dynamics of our model are not analytically tractable, we constructed adaptive landscapes numerically. We initialized models with populations fixed for a S allele conferring choosinesses *a*_*S*_ and *b*_*S*_ in females and males, respectively. We initialized one model for every combination of *a*_*S*_ *, b*_*S*_ ∈ exp({0, 0.5, …, 10}). Starting with the t allele rare, we iterated generations until the T and t alleles reached mutation–selection balance. We called the population at mutation–selection balance the “resident population.” Into the resident population, we introduced a low frequency (0.001) of a mutant s allele conferring choosinesses *a*_*s*_ and *b*_*s*_. We considered cases in which (*a*_*s*_, *b*_*s*_) = (*a*_*S*_ + 0.01, *b*_*S*_) and in which (*a*_*s*_, *b*_*s*_) = (*a*_*S*_, *b*_*S*_ + 0.01). That is, we considered mutations that affect just female or just male choosiness, but not mutations that affect both. To estimate the invasion fitness of each mutant, we iterated 1000 generations and we asked whether and how fast the mutant increased or decreased in the population. In large populations, the probability that a rare mutant allele replaces a resident allele is proportional to its fitness (Fisher, 1930). Therefore, we assumed that the direction of evolution in phenotype space is proportional to the fitness of mutant alleles affecting female and male imprinting. This is reasonable if mutations affecting female and male choosiness are small and occur at the same rate.

**Figure 2:**
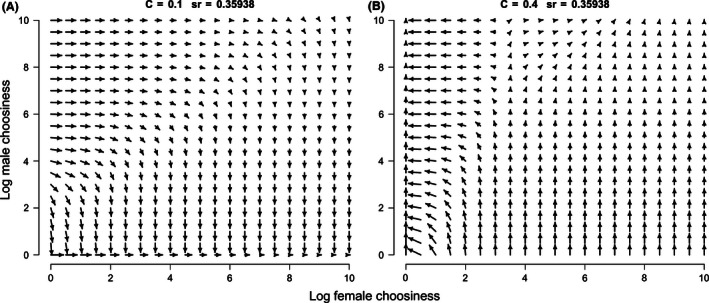
Adaptive landscapes when females have paternal imprinting and males have maternal imprinting, α = 0.36 and (A) *c *=* *0.1 or (B) *c *=* *0.4. Arrows show the direction of selection, pointing to adaptive peaks. The length of each arrow is proportional to the strength of selection. The origin of each panel represents random mating. In (A), strong choosiness by females (bottom right corner) evolves from random mating. In (B), strong choosiness by males evolves from random mating (top left corner). All adaptive landscapes are archived at…Dryad (doi:10.5061/dryad.hn082)

Figure 2 shows representative adaptive landscapes when α = 0.36, the cost of courtship is 0.1 (A) or 0.4 (B), and females imprint on their fathers but males imprint on their mothers. Locally stable choosinesses can be classified into two types. The first type is the bottom‐up ESS, which evolves by a series of small mutations from random mating. Strong paternal imprinting by females in Fig. 2A is an example. The second type is the alternative ESSs, which cannot evolve from random mating, but can evolve if strong imprinting already exists in the population. Strong imprinting by both sexes in Fig. 2B is an example.

### 3.2 Finding globally stable imprinting strengths

For each combination of courtship cost and sex ratio, we analyzed the set of locally stable imprinting strategies to identify those that cannot be invaded by strategies with the same modes and any other choosinesses. We call these strategies within‐mode ESSs. To test whether locally stable choosinesses are within‐mode ESSs, we initialized population dynamics models with populations fixed for a S allele that conveys the locally stable choosiness. We iterated generations until the T and t alleles reached mutation–selection balance. Into this resident population, we introduced a low frequency (0.001) of a mutant s allele that confers choosinesses (*a*_*s*_ *, b*_*s*_), and we iterated 1000 more generations. If the mutant increased in frequency, we said it invaded the resident. We considered all mutants with *a*_*s*_ *, b*_*s*_ ∈ exp({0, 0.5, …, 10}). Only locally stable strategies that were not invaded by any of the mutant alleles are within‐mode ESSs.

**Figure 3:**
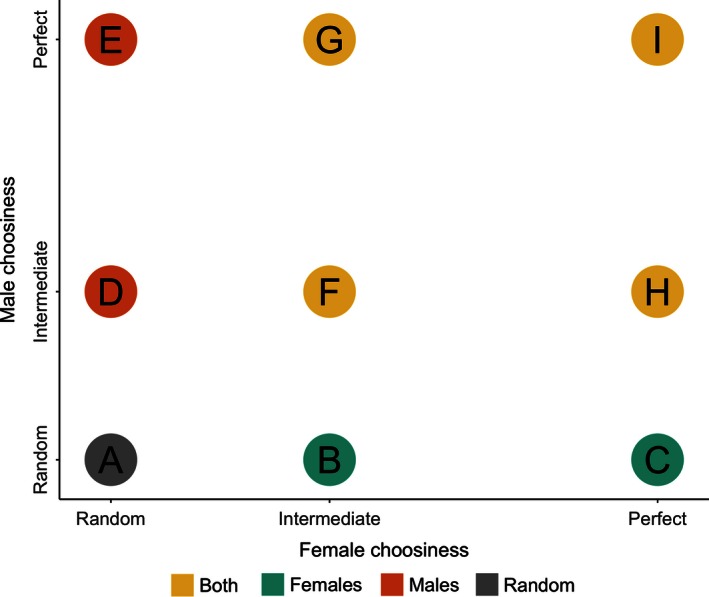
Schematic showing qualitatively different within‐mode evolutionary stable strategies types. The *x*‐axis shows choosiness by females, and the *y*‐axis shows choosiness by males. Intermediate refers to any choosiness that is neither random nor perfect. Random mating (gray) can be a stable state (A). Female‐only imprinting (blue) can evolve to intermediate (B) or perfect choosiness (C). Male‐only imprinting (orange) can evolve to intermediate (D) or perfect choosiness (E). Imprinting by both sexes (yellow) can evolve to intermediate choosiness in both sexes (F), perfect choosiness in males and intermediate in females (G), intermediate choosiness in males and perfect in females (H), or perfect choosiness in both sexes (I)

We classified choosinesses at each within‐mode ESSs into one of three categories: random mating (i.e., individuals never reject potential mates), intermediate (i.e., individuals are choosy, but choosiness is less than the maximum strength we tested), and perfect (i.e., choosiness evolves to the maximum strength we tested). Thus, there are nine qualitatively different types of within‐mode ESSs (Fig. 3). In type A ESSs, both females and males mate randomly. These can be stable under any combination of female and male imprinting modes. In type B and D ESSs, one sex evolves intermediate choosiness and the other mates randomly. These occur when the choosy sex exhibits oblique or same‐sex imprinting (i.e., females imprint on their mothers or males imprint on their fathers). In type C ESSs, females evolve perfect choosiness and males mate randomly. This occurs only when females imprint on their fathers. In type E ESSs, males evolve perfect choosiness and females mate randomly. This occurs only when males imprint on their mothers. In ESS types F to I, both sexes evolve choosiness. In type F ESSs, both sexes evolve intermediate choosiness. This occurs when males imprint obliquely and females imprint either obliquely or on their mothers. In type G ESSs, females evolve intermediate choosiness and males evolve perfect choosiness. This occurs when females imprint obliquely and males imprint on either parent. In type H ESSs, females evolve perfect choosiness and males evolve intermediate choosiness. This occurs when females imprint on their fathers and males imprint obliquely. Finally, in type I ESSs, both sexes evolve perfect choosiness. This occurs when both sexes imprint on their parents.

To understand why the evolutionarily stable choosiness depends on the imprinting mode, consider oblique imprinting by females when mating opportunities are not limiting (i.e., every female eventually mates). The proportion of females imprinted on the fitter T allele is equal to the proportion of adult males in the population carrying that allele. If mating is random, the proportion of females that accept males with the T allele is the proportion of T males in the population. If choosiness is perfect, females never accept nonpreferred males, and the proportion of females that accept T males is the same. However, if choosiness is intermediate, most females that have imprinted on the common T allele find and accept T males, but some females that have imprinted on the t allele fail to find t males and accept T males instead. So, intermediate choosiness maximizes the chance that females obtain T mates. A similar argument holds for oblique imprinting by males and for imprinting on the same‐sex parent by either sex. Imprinting on the opposite‐sex parent is different. In this case, as choosiness increases, individuals with the less fit t allele are rarely chosen as mates, and so rarely enter the imprinting set (i.e., opposite‐sex parents). As the t allele becomes rarer in the imprinting set, the optimal choosiness (i.e., the choosiness that maximizes the probability of finding a T mate) increases. The optimal choosiness is always greater than the current choosiness, so choosiness evolves toward perfection and eliminates the t allele from the imprinting set.

If both sexes imprint on their parents, then perfect choosiness evolves even when imprinting is on the same‐sex parent. In this case, choosiness by one sex eliminates the t allele from the imprinting set of the other. Interestingly, oblique imprinting by females allows paternally imprinting males to evolve perfect choosiness, but oblique imprinting by males does not allow maternally imprinting females to evolve perfect choosiness. This is because sexual selection imposed by females is stronger than sexual selection imposed by males. Males can mate multiple times, so when females are choosy, almost all males selected as mates have the T phenotype, and the t phenotype is eliminated from the paternal imprinting set. In contrast, each female can only mate once, so even rare matings by females with the t allele maintain the t allele in the maternal imprinting set.

**Figure 4:**
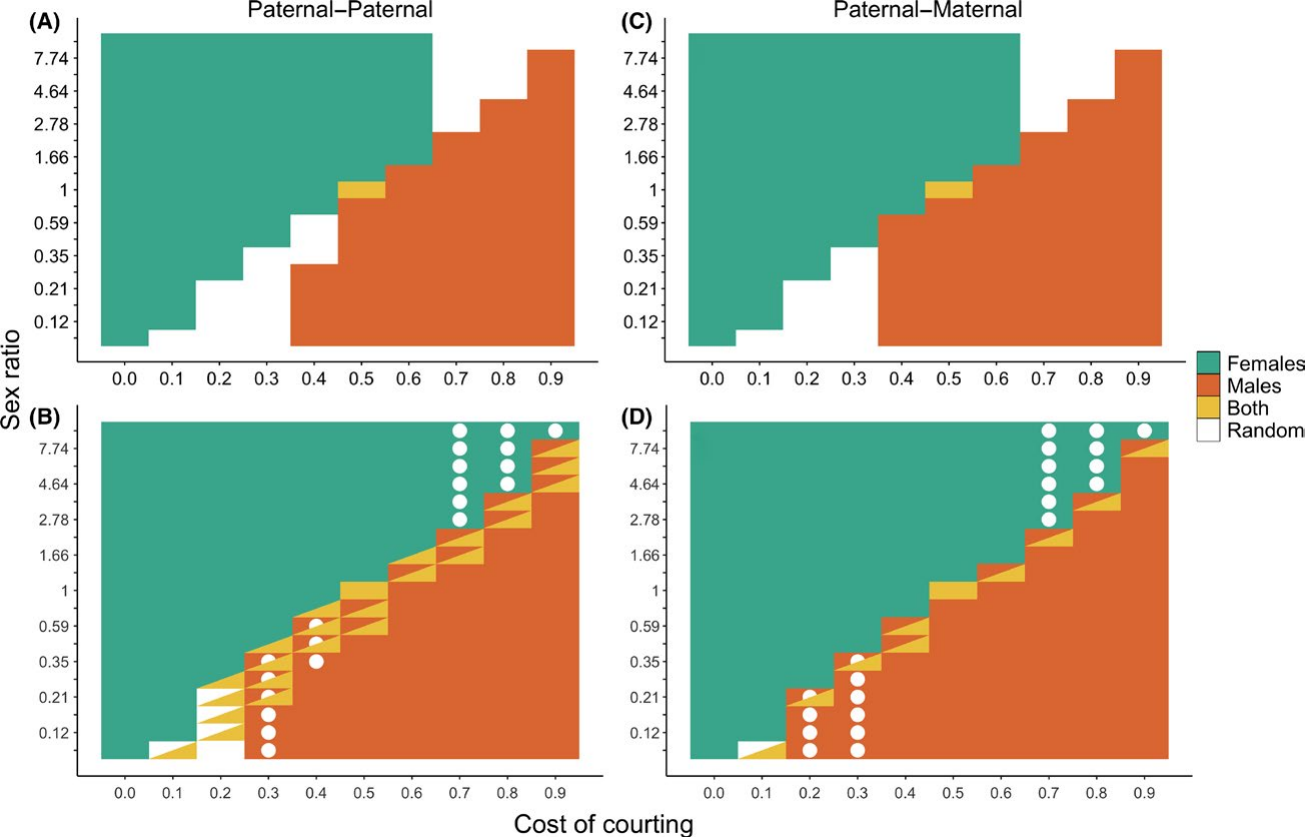
Combinations of courtship cost (*x*‐axis) and sex ratio (*y*‐axis) under which sexual imprinting by females (blue), males (orange), or both sexes (yellow) is evolutionarily stable. (A and B) show within‐mode evolutionary stable strategies (ESSs) when both sexes imprint paternally. (C and D) show within‐mode ESSs when females imprint paternally and males imprint maternally. (A and C) show bottom‐up ESSs, and (B and D) show all possible ESSs. In boxes with two colors, there are two possible stable states, and in those with two colors and a dot, there are three possible stable states

Figure 4 shows the combinations of sex ratio and courtship cost under which imprinting by females, males, or both sexes can evolve when both sexes imprint on their fathers (A, B) or when females imprint on their fathers and males imprint on their mothers (C, D). Results under other combinations of imprinting modes are shown in Appendix 4. Panels A and C show the bottom‐up ESSs, and B and D show all the possible within‐mode ESSs.

In general, sexual imprinting by females evolves when the cost of courtship is less than the proportion of males in the adult population (i.e., *c *< α/(1 + α)). When this is true, mating opportunities for females are not limiting. Some males remain in the mating pool after all females have mated, and it is advantageous for females to choose only the best males as mates. Sexual imprinting by males evolves when the cost of courtship is greater than the proportion of males in the population (i.e., *c *> α/(1 + α)). When this is true, reproductive success for males is limited not by the availability of females but rather by the number of courtships males can perform. Thus, it is advantageous for males to allocate courtships only to the most valuable females. In a narrow sliver of parameter space close to *c *= α/(1 + α), imprinting by both sexes can evolve.

### 3.3 Between‐mode evolutionary stable states

Some within‐mode ESSs may be invasible by imprinting strategies with different modes. To determine whether a within‐mode ESSs is stable against invasion by other modes, we conducted invasion analyses with the resident population at the within‐mode ESS. We assumed that the imprinting mode and choosiness are controlled by different loci and that mutations at these loci are sufficiently rare that each mutation is fixed or eliminated before a new one arises. Thus, if the S and s alleles differ in their mode of imprinting, they do not differ in their choosiness, and each sex is monomorphic for choosiness while different imprinting modes compete for fixation. This means that we only needed to consider invading imprinting modes at the ESS strength of the resident mode, which simplifies our analysis.

Into the resident population at its within‐mode ESS, we introduced a low frequency (0.001) of a mutant s allele that confers a different imprinting mode but the same choosiness as the resident. We introduced the mutant in linkage equilibrium with the target trait allele. We tested the female and male imprinting modes separately for stability against invasion by each other imprinting mode. Thus, each ESS was tested against four possible invaders: two with different modes of imprinting by females and two with different modes of imprinting by males. We iterated 1000 generations, and we asked whether the mutant increased or decreased in the population. Only within‐mode ESSs that were not invaded by any other mode combination are between‐mode ESSs. The between‐mode ESS is the strategy we expect to see in nature.

**Figure 5:**
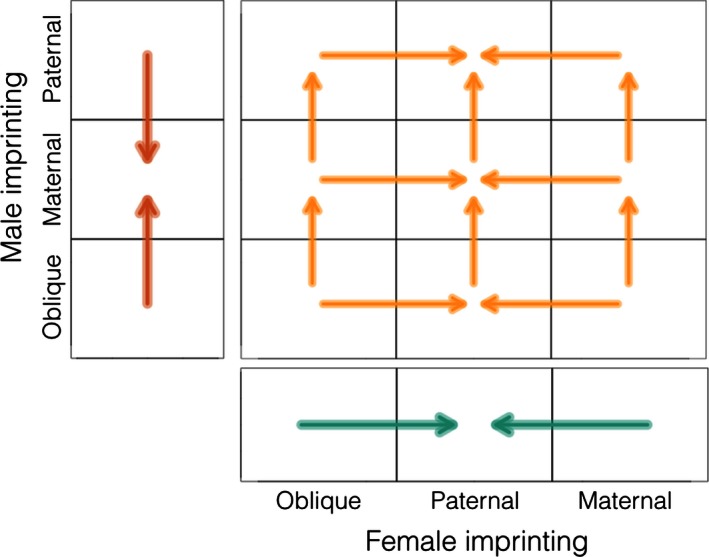
Schematic figure of the evolutionary trajectory of imprinting modes. The mode or mode combination at the base of each arrow can be replaced by the mode or mode combination at the head. When sexual imprinting is by males, maternal imprinting can invade and replace each of the other modes (orange arrows). When sexual imprinting is by females, paternal imprinting can invade and replace each of the other modes (blue arrows). When imprinting occurs in both sexes, the imprinting modes of each sex are invaded and replaced until both sexes imprint paternally (yellow arrows)

Figure 5 is a schematic showing the direction of evolution of the different imprinting modes (i.e.*,* which imprinting modes or mode combinations can invade which other modes or mode combinations). The mode or mode combination at the base of each arrow can be replaced by the mode or mode combination at the head. When sexual imprinting by only males evolves, maternal imprinting can invade and replace each of the other modes (orange arrows). When sexual imprinting by only females evolves, paternal imprinting can invade and replace each of the other modes (green arrows). When imprinting by both sexes evolves, imprinting modes of each sex are invaded and replaced until both sexes imprint paternally (gold arrows). Thus, imprinting on the opposite‐sex parent is always the stable strategy, except when both sexes imprint. In this case, paternal imprinting by both sexes is the stable strategy.

**Figure 6:**
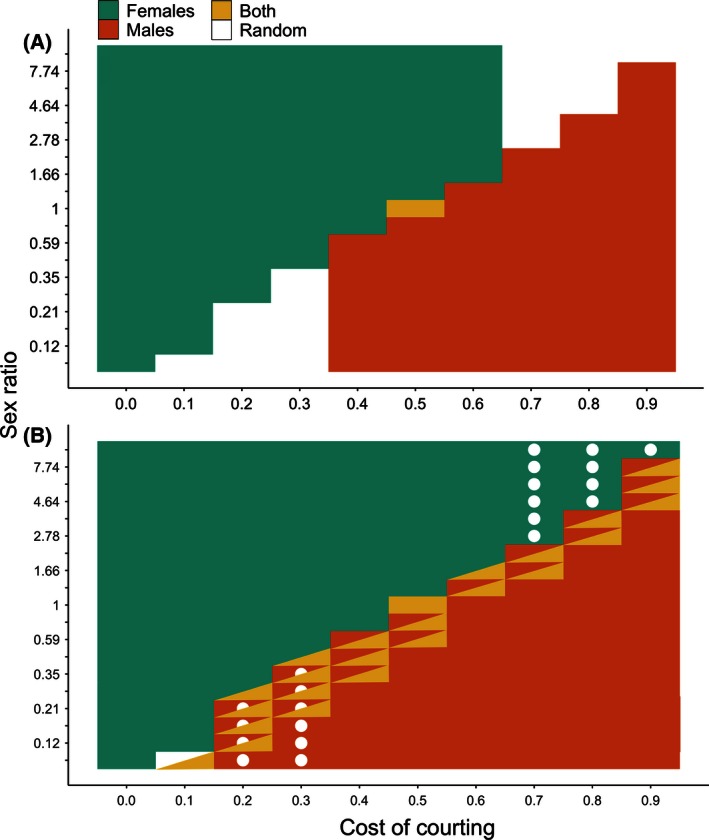
Choosy sexes at between‐mode evolutionary stable strategies (ESSs). Axes show cost of courtship (*x*‐axis) and sex ratio (*y*‐axis). Blue represents female sexual imprinting, orange represents male sexual imprinting, yellow represents imprinting by both sexes, and white represents random mating. (A) shows the between‐mode ESSs that evolve from random mating, and (B) shows all possible between‐mode ESSs. In boxes with two colors, there are two stable states, and in boxes with two colors and a dot, there are three stable states

Figure 6 shows the choosy sex at the between‐mode ESS for different combinations of sex ratio and courtship cost. Panel A shows ESSs that evolve from random mating, and panel B shows all possible between‐mode ESSs. When the cost of courtship is low, females evolve imprinting and paternal imprinting can invade and replace any other mode. When the cost of courtship is high, male imprinting evolves and maternal imprinting is stable. For a small range of intermediate costs, both males and females can evolve imprinting and both sexes evolve to imprint paternally.

## 4 Discussion

Past theoretical work has examined the conditions under which sexual imprinting by females evolves (Chaffee et al., 2013; Invernizzi & Gilman, 2015; Tramm & Servedio, 2008). Here, we study the conditions under which sexual imprinting by males evolves. Both male and female imprinting can evolve in our model, but they rarely evolve under the same conditions. Thus, imprinting by both sexes in the same population is rare. If sexual imprinting by males evolves, maternal imprinting is the most advantageous strategy, and if sexual imprinting by females evolves, paternal imprinting is the most advantageous strategy. In the rare cases where both sexes evolve imprinting, the most advantageous strategy is for both sexes to imprint on their fathers.

The evolution of sexual imprinting is influenced by the operational sex ratio and the cost of courtship. In general, if the operational sex ratio is strongly biased, members of the rarer sex have many mating opportunities and can afford to be choosy. In contrast, members of the more common sex have limited mating opportunities and must take those opportunities even when potential mates are of low quality. Courtship costs favor the evolution of male choosiness. Costs limit the number of courtships a male can achieve, and when his ability to court is limited, he should court only high‐quality females. Courtship costs also reduce the number of males in the mating pool. This reduces mating opportunities for females, and inhibits the evolution of female choosiness. Thus, the conditions that increase selection for choosiness in one sex inhibit selection for choosiness in the other.

Imprinting by both sexes can evolve from random mating only when the costs of courtship are high and males are common. Under these conditions, female can expect to have a large numbers of mating opportunities and female choosiness can evolve. Choosiness by females increases the strength of selection acting on males. Once females become sufficiently choosy, males are likely to be rejected if they court females with phenotypes different from their own. It is not worth investing in costly courtship if the probability of rejection is high. So, choosiness by females promotes the evolution of male choosiness.

In nature, imprinting by both sexes may not always have to evolve from random mating. For example, consider a system in which the operational sex ratio is 0.36 and the cost of courtship is 0.1 (Fig. 2A). In this case, we expect the evolution of strong choosiness by females and random mating by males. If the cost of courtship increases to 0.4, perhaps due to reduced resource availability or increased predation on courting males, then strong choosiness by females and random mating by males is no longer evolutionarily stable. We would expect imprinting by both sexes to evolve from this starting point (Fig. 2B). Environmental change may affect costs of courtship and sex ratios in many species. For example, in species with temperature‐dependent sex determination (TSD), climate change can affect sex ratios, skewing them toward females (Janzen, 1994) or males (Ospina‐Alvarez & Piferrer, 2008). Environmental change can also affect sex ratios in species without TSD. For example, red deer (*Cervus elaphus*) produce fewer male offspring under climatic conditions that induce nutritional stress (Kruuk et al., 1999; Mysterud et al., 2000). Male‐biased hunting by humans can also skew animal populations toward females (*e.g*., impalas, Setsaas et al., 2007; African lions, Loveridge et al., 2007; bycatch of Galapagos waved albatross, Awkerman et al., 2006). Habitat loss and fragmentation have been related to increased predation risk (Crooks & Soulé, 1999; Keller & Waller, 2002), which can increase the costs of courtship in males. Environmental changes like these might allow alternative ESSs to evolve via multistep processes.

Kokko and Monaghan (2001) predicted that choosiness should evolve in the sex with the higher cost of breeding, where the cost of breeding is defined as a reduced ability to invest in future offspring. In our model, females always experience a high cost of breeding, because after breeding once, they have no further reproductive potential. Males experience a high cost of breeding only if courtship is costly. Female choosiness evolves when the cost of courtship for males is low, and male choosiness evolves only when the cost of courtship is high. Kokko and Monaghan (2001) also predicted that mutual mate choice would rarely evolve, and we obtained a similar result. Thus, our results show that Kokko and Monaghan's predictions hold when the mate preference is not for a specific trait but rather for an imprinted phenotype. Consequently, we have expanded their results to a new class of mate choice behavior. Moreover, our model expands Kokko and Monaghan's results by predicting not just when choosiness will evolve, but also how strong choosiness will become and what choosiness strategies will be used.

Previous studies of sexual imprinting by females suggest that imprinting on the opposite‐sex parent is the stable strategy (Chaffee et al., 2013; Tramm & Servedio, 2008). Here, we show that imprinting on the opposite‐sex parent is also stable when males are the choosy sex. Cross‐sex imprinting is advantageous because parents of the chosen sex have survived both viability and sexual selection, and as a result are more likely to carry favorable alleles than parents of the choosy sex. Thus, the opposite‐sex parental phenotype is an accurate indicator (*i.e*., an honest signal) that a potential mate will produce fit offspring.

If imprinting evolves in both sexes, the stable strategy is for both sexes to exhibit paternal imprinting. This is true because males can mate multiple times but females cannot. This means that variability in male reproductive success is higher than variability in female reproductive success and that sexual selection acts more strongly on males than on females. Because fathers have been successful in this strong sexual selection, they are more likely than mothers to carry favorable alleles. This makes it advantageous for members of both sexes to imprint on their fathers.

The evolution of sexual imprinting by males in our model requires high courtship costs. In nature, courtship costs are high for animals that engage in sexual cannibalism. In the praying mantis *Pseudomantis albofimbriata*, the probability that a courting male is cannibalized may be as high as 90% depending on the condition of the female (Barry et al., 2008). Some spiders also experience high courtship costs (Elgar, 1992). In vertebrates, courtship costs are believed to be much lower (Jordan & Brooks, 2010). Monogamous systems may be exceptions. Males that enter or attempt to enter into monogamous partnerships may forgo many other courtship and mating opportunities. Models of polygynous systems, like the one presented here, may not accurately predict the evolution of sexual imprinting in monogamous systems (Invernizzi & Gilman, 2015). More work is needed to understand how imprinting by males might evolve in such systems.

Our analysis of between‐mode ESSs assumes that choosiness can be redirected from a maternal to a paternal phenotype (or vice versa) by a single mutation. We do not know whether this happens in nature. In fact, little is known about the genetics of sexual imprinting. However, it seems plausible that an existing preference might be redirected more easily than a new preference could evolve. An alternative assumption would be that a strong preference for one parent's phenotype must first be replaced by a very weak preference (*i.e*., nearly random mating) for the phenotype of the other and that this preference could then evolve to become strong. In our model, within‐mode ESSs resist invasion by random mating. Thus, if preferences cannot be easily redirected between parental phenotypes, then the imprinting modes that evolve first in a given system may be more stable than our analysis suggests.

In this study, our goal was to focus on the evolution of different sexual imprinting strategies. Therefore, we excluded genetically inherited mate preferences from our model. The evolution of genetic preferences has been studied extensively elsewhere (van Doorn et al., 2009; Servedio & Bürger, 2014; Veen & Otto, 2015). If a genetic preference for the T phenotype were available in our model, it would be the most accurate way for individuals to identify fit mates, and we would expect it to evolve more readily than imprinting. Given the apparent advantage of genetic over imprinted preferences, why sexual imprinting is such a common strategy in nature remains an open question.

Our results provide novel predictions about the imprinting modes and imprinting strengths we should expect to see in nature. However, our understanding of how sexual imprinting evolves is still in the early stages. Sexual imprinting may be involved in sex identification, species identification, or in the identification of mates that provide good parental care rather than simply good genes as in our model. Theory for the role of these drivers in promoting the evolution of sexual imprinting has not been formalized. Additional models and empirical tests of sexual imprinting across a range of species will allow us to advance our existing knowledge.

## Funding Information

Natural Environment Research Council, (Grant/Award Number: “NE/K500859/1”).

## Conflict of Interest

None declared.

## Figures and Tables

**Figure A1:**
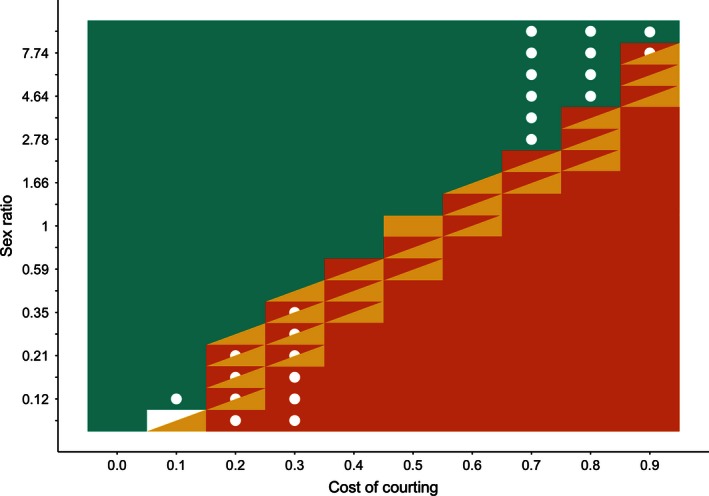
All possible between‐mode ESSs when viability selection on the target trait acts only in males. Axes show cost of courtship (*x*‐axis) and sex ratio (*y*‐axis). ESSs are imprinting by females (blue), males (orange), both sexes (yellow), or random mating (white). When random mating is stable, random mating cannot be invaded by any imprinting strategy, and we do not expect imprinting to evolve de novo

**Figure A2:**
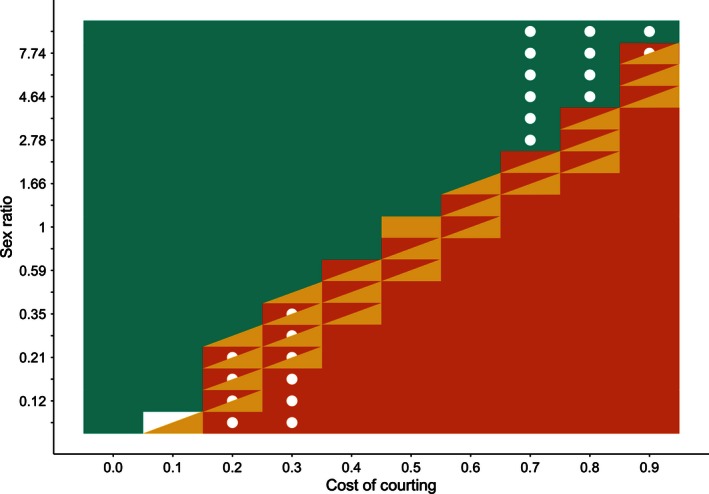
All possible between‐mode ESSs when viability selection on the target trait is strong (*v *=* *0.9) and sexually monomorphic. Axes show cost of courtship (*x*‐axis) and sex ratio (*y*‐axis). ESSs are imprinting by females (blue), males (orange), both sexes (yellow), or random mating (white). When random mating is stable, random mating cannot be invaded by any imprinting strategy, and we do not expect imprinting to evolve de novo

**Figure A3:**
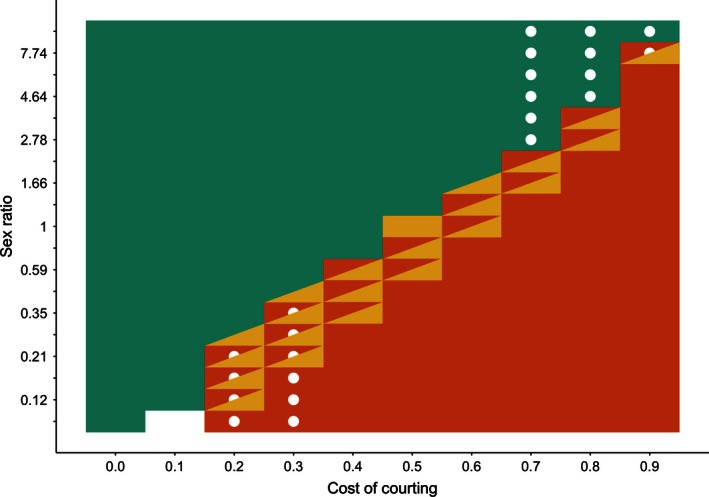
All possible between‐mode ESSs when encounter rates during mating are density dependent. Axes show cost of courtship (*x*‐axis) and sex ratio (*y*‐axis). ESSs are imprinting by females (blue), males (orange), both sexes (yellow), or random mating (white). When random mating is stable, random mating cannot be invaded by any imprinting strategy, and we do not expect imprinting to evolve de novo. To see how encounter rate affects the parameter space under which imprinting evolves, compare areas with white or white dots in this figure to areas with white or white dots in Fig. 6B

**Figure A4:**
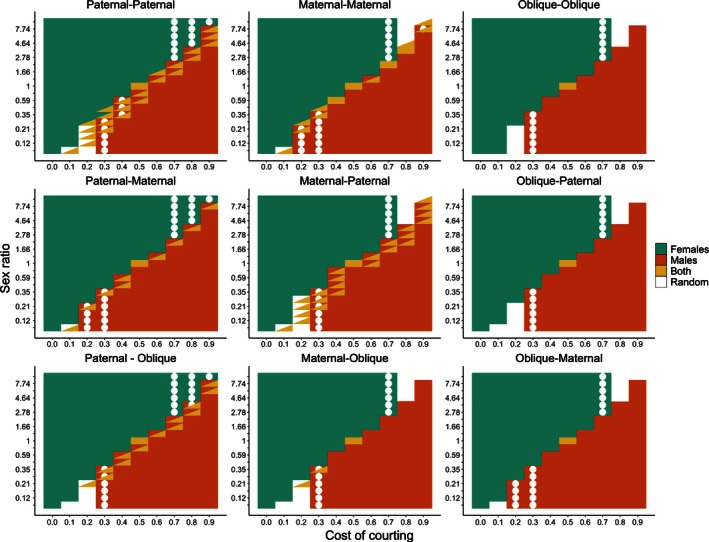
Within‐mode ESSs for all nine combinations of imprinting modes for females and males. Axes show cost of courtship (*x*‐axis) and sex ratio (*y*‐axis). Titles show the combination of imprinting modes in the format (females–males). ESSs are imprinting by females (blue), males (orange), both sexes (yellow), or random mating (white)

## Appendix 1

### Evolution of sexual imprinting when viability selection acts on the target trait in males but not in females.

In the body of the study, we studied scenarios in which viability selection on the target trait is sexually monomorphic. In nature, many traits are differently expressed in males and females, and thus, viability selection may differ between the sexes. Here, we studied viability selection that acts on the target trait in males but not in females. We implemented this by setting *v*_*t*_ = 0.01 for males and *v*_*t*_ = 0 for females in eq. (1). Results are qualitatively similar to those reported in the body of the study (Fig. A1). In particular, greater mate availability promotes the evolution of sexual imprinting by each sex. Imprinting on the opposite‐sex parent is the stable strategy when only one sex evolves imprinting. When both sexes evolve imprinting, paternal imprinting is the stable strategy for both sexes.

## Appendix 2

### Evolution of sexual imprinting when viability selection on the target trait is strong.

In the body of the study, we studied the evolution of sexual imprinting when viability selection on the target trait is weak (*i.e*., *v*_*t*_ = 0.01). Here, we report between‐mode ESSs for sexual imprinting when viability selection on the target trait is strong (*i.e*., *v*_*t*_ = 0.9). Results are qualitatively similar to those reported in the body of the study.

## Appendix 3

### Evolution of sexual imprinting when encounter rates between the sexes are density dependent.

In the body of this study, we assumed that every member of the rarer sex in the mating pool meets a potential mate in every round of mating. Thus, the encounter rate depends on the sex ratio but not on the density of individuals in the mating pool. In nature, encounter rates may decrease when densities of available males and females are low. Lower encounter rates mean fewer mating opportunities, and fewer mating opportunities inhibit the evolution of sexual imprinting. We demonstrate that in this appendix. Here, we replace eq. (3) with:

(A3) $$E_{xyz}^{ijk}\left(r\right)=M_{ijk}^{\prime \prime }\left(r\right)F_{xyz}^{\prime }\left(r\right)$$

Thus, the density of encounters between males with phenogenotype *ijk* and females with phenogenotype *xyz* in round *r* of mating is proportional to the density of the male and female phenogenotypes in the mating pool in that round. Results are qualitatively similar to those reported in the body of the study (Fig. A3), but the parameter space in which sexual imprinting evolves is slightly reduced.

## Appendix 4

### Within‐mode ESSs for all combinations of female and male imprinting modes.

In the body of this study, we presented within‐mode ESSs for two combinations of female and male imprinting modes: paternal(f)–maternal(m) and paternal(f)–paternal(m). Here, we report the within‐mode ESSs for all combinations of female and male imprinting modes (Fig A4).
